# Estradiol and zinc-doped nano hydroxyapatite as therapeutic agents in the prevention of osteoporosis; oxidative stress status, inflammation, bone turnover, bone mineral density, and histological alterations in ovariectomized rats

**DOI:** 10.3389/fphys.2022.989487

**Published:** 2022-09-19

**Authors:** Mona M. Elghareeb, Gehad E. Elshopakey, Tarek A. Elkhooly, Basma Salama, Alaa Samy, Fuller W Bazer, Mohammed A Elmetwally, Mikhlid H. Almutairi, Lotfi Aleya, Mohamed M. Abdel-Daim, Shaymaa Rezk

**Affiliations:** ^1^ Department of Physiology, Faculty of Veterinary Medicine, Mansoura University, Mansoura, Egypt; ^2^ Department of Clinical Pathology, Faculty of Veterinary Medicine, Mansoura University, Mansoura, Egypt; ^3^ Nanomedicine Research Unit, Faculty of Medicine, Delta University for Science and Technology, Belqas, Egypt; ^4^ Refractories, Ceramics, and Building Materials Department, National Research Centre, Giza, Egypt; ^5^ Department of Physics, Faculty of Science, New Mansoura University, New Mansoura City, Egypt; ^6^ Department of Biochemistry and Chemistry of Nutrition, Faculty of Veterinary Medicine, Mansoura University, Mansoura, Egypt; ^7^ Department of Surgery, Anesthesiology, and Radiology, Faculty of Veterinary Medicine, Mansoura University, Mansoura, Egypt; ^8^ Department of Animal Science, Texas A&M University, College Station, TX, United States; ^9^ Department of Theriogenology, Faculty of Veterinary Medicine, Mansoura University, Mansoura, Egypt; ^10^ Department of Zoology, College of Science, King Saud University, Riyadh, Saudi Arabia; ^11^ UMR CNRS 6249, Chrono-Environnement Laboratory, Bourgogne, Franche-Comté University, Besançon, France; ^12^ Pharmacology Department, Faculty of Veterinary Medicine, Suez Canal University, Ismailia, Egypt; ^13^ Department of Cytology and Histology, Faculty of Veterinary Medicine, Mansoura University, Mansoura, Egypt

**Keywords:** Osteoporosis, estradiol, zinc-doped nano hydroxyapatite, oxidative/inflammatory markers, PINP/VEGF/PCNA

## Abstract

Osteoporosis (OP) is a serious health problem, and the most popular therapeutic strategy for OP is hormone replacement (estrogen); however, it increases the risk of reproductive cancers. Hydroxyapatite (HA) nanoparticles have a similar chemical structure to the bone mineral component and can be used as a new remedy for OP. This study was designed to investigate the osteoporosis-protective potential of nano zinc hydroxyapatite (ZnHA-NPs) and/or estradiol (E2) combined therapy. A total of 35 adult female rats were assigned into five groups (*n* = 7): 1) control group; 2) ovariectomized group (OVX); 3) OVX received oral estradiol replacement therapy (OVX/E2); 4) OVX received ZnHA replacement therapy (OVX/ZnHA); and 5) OVX received both estradiol and ZnHA-NPs combined therapy (OVX/E2+ZnHA). After 3 months of treatment, serum bone markers and estrogen level, oxidative/antioxidant, and inflammatory cytokines were determined. Additionally, femoral expression of estrogen receptors alpha and beta (ESR1; ESR2), receptor activator of nuclear factor-kappa B (RANKL) ligand, osteoprotegerin (OPG), bone mineral density (BMD), histological alterations, and immunohistochemical expression of vascular endothelial growth factor (VEGF) and proliferating cell nuclear antigen (PCNA) were assessed. ALP, PINP, Ca, and P concentrations improved significantly (*p* < 0.05) in all treatment groups, especially in the OVX/E + ZnHA group. MDA and NO were higher in OVX rats, while SOD activity and GSH were lower (*p* < 0.05). E2 alone or with ZnHA-NPs restored the estimated antioxidant molecules and cytokines toward normal levels in OVX rats (*p* < 0.05). On the other hand, E2 and ZnHA increased OPG and OC expression in femurs while decreasing ESR1, ESR2, and NF-kB expression (*p* < 0.05). The combination treatment was superior in the restoration of normal femoral histoarchitecture and both cortical and trabecular BMD (*p* < 0.05). Overall, the combined therapy of OVX/E2+ZnHA was more effective than the individual treatments in attenuating excessive bone turnover and preventing osteoporosis.

## Introduction

Osteoporosis (OP) is one of the most serious health problems, with rates steadily increasing in recent decades. It is characterized by an abrupt loss of organic matrix and minerals as a result of a decrease in osteoblast activity and an increase in osteoclasts. Bone fragility and fractures develop as bone mass diminishes (Nasonov, 2011). By 2050, the global incidence of osteoporosis-related hip fractures is expected to rise by around 310 %t in men and 240% in women (Gullberg et al., 1997).

Based on the evidence of effectiveness, cost, and safety, standard HRT should be considered one of the first-line therapies for the prevention and treatment of fractures in postmenopausal women younger than 60 years (Cobin et al., 2017; Levin et al., 2018). Hormone replacement therapy (HRT) in the form of either combined estrogen and progesterone or estrogen alone is effective in reducing the number of both vertebral and non-vertebral fractures in postmenopausal women (Jin et al., 2017). Thus, standard HRT is effective in preventing bone loss associated with menopause and decreases the incidence of all osteoporosis-related fractures, including vertebral and hip fractures (De Villiers and Stevenson, 2012). Estrogen is a steroid hormone that is essential for bone formation and homeostasis. After menopause, estrogen levels in females drop, causing bone loss by increasing both osteoclast numbers and activity (Hofbauer et al., 2000), leading to receptor activator of nuclear factor-kappa B (NF-kB) ligand-osteoprotegerin (RANKL-OPG) imbalance (Khosla et al., 2011). In addition, estrogen is known to play a significant role in angiogenesis. As a result, hormone replacement therapy (HRT) has been a popular therapeutic strategy designed for postmenopausal osteoporosis (Prelevic et al., 2005). However, long-term HRT may increase the risk of cancer in reproductive tissues (Gray, 2003). Thus, low dose-HRT may enhance patient continuation, with adequate bone protection and menopausal symptom control. The beneficial effects of the low-dose regimens of HRT are minimizing vasomotor symptoms and vaginal atrophy, lipid profiles, bleeding profiles, and endometrial hyperplasia (Smith et al., 2014).

Nowadays, researchers have turned to nanomaterials to develop novel and alternative osteoporosis treatments that reduce systemic toxicity and improve chemical drug therapeutic efficacy (Ferbebouh et al., 2021). Hydroxyapatite (HA) is a major chemical constituent of skeletal bone, and it is converted into Ca^2+^ and PO_4_^3-^ in the body (Couto et al., 2010). When utilized in orthopedics, HA promotes bone development and osteointegration, possibly by increasing adhesion and osteoblast proliferation, resulting in faster healing of bone (Kamitakahara et al., 2014). Therefore, HA promotes osteoblast bioactivity to speed-up bone regeneration (Lin et al., 2009; Iafisco et al., 2014). In addition, HA is commonly used in bone repair as bone addition, coating implants, and bone fillers (Zhou and Lee, 2011). The main advantages of hydroxyapatite are biocompatibility, low *in situ* biodegradation, and good osteoconduction (Kee et al., 2013). Nonetheless, hydroxyapatite nanocrystal is difficult to form the specific formula needed for bone repair and implantation. This is due to the intrinsic hardness, fragility, and lack of flexibility, thereby restricting the use of load-bearing implant material (Sun et al., 2011). Therefore, hydroxyapatite nanocrystal is often combined with various polymers to produce osteoconductive biocomposite material in the field of orthopedic surgery (Zhou and Lee, 2011).

The calcium present in HA can be exchangeable for metal ions such as zinc, magnesium, and strontium (Mora Raimundo et al., 2017). Zn enhances the bioactivity of HA (Ito et al., 2005; Khajuria et al., 2018) since zinc-containing tricalcium phosphate is indicated to promote osteogenesis in osteoporotic bone (Tokudome et al., 2011; Chou et al., 2013), compared with HA (Bhattacharjee et al., 2014). Because Zn has characteristics similar to calcium, it can be used to replace calcium in HA in bone (Khajuria et al., 2016; Mora Raimundo et al., 2017). Furthermore, Zn itself has a very essential role in the maintenance of the skeletal system. The cellular activities of osteoblasts and osteoclasts are known to be influenced positively by zinc-related proteins; therefore, it has the potential to slow bone deterioration in the early stages of bone loss (Bhardwaj et al., 2016). Additionally, zinc may have a role in the secretion of estrogen from glands other than the ovary (Bhardwaj et al., 2016).

Thus, the main purpose of our study was to assess the prospective roles of nanoparticles of zinc-doped hydroxyapatite (ZnHA-NPs) to prevent or treat osteoporosis in comparison to E2 administration through the evaluation of serum bone turnover markers and oxidative/inflammatory responses, femoral expression of RANKL-OPG, vascular endothelial growth factor (VEGF), and proliferating cell nuclear antigen (PCNA). In addition, our study aimed to lower estrogen dose to prevent its side effects by combining it with ZnHA-NPs.

## Material and methods

### Chemicals

Estradiol (E2); estradiol valerate tablets (pyrgynova^@^ 2 mg), an original research product, were obtained from Bayer Zydus Pharma Company (Germany). Zinc-doped hydroxyapatite nanoparticles (ZnHA-NPs); calcium nitrate tetrahydrate (Ca(NO_3_)_2_4H_2_O), diammonium hydrogen phosphate ((NH4)2HPO4), and zinc nitrate hexahydrate Zn(NO3)6 6H2O were purchased from Sigma-Aldrich Company (United States).

### Preparation and characterization of zinc-doped hydroxyapatite nanoparticles

ZnHA nanoparticles were prepared by the acid/base precipitation method according to several previous publications (Popa et al., 2016; Predoi et al., 2017). In brief, both calcium and zinc nitrates were mixed and dissolved in 500 ml of distilled water (D.W), and zinc concentrations were determined to replace calcium in stoichiometric hydroxyapatite lattice by adjusting Zn/(Ca + Zn) equal to 1 mol%. Then, the solution was heated to 80 ^o^C, and an appropriate concentration of diammonium hydrogen phosphate was added dropwise to the mixture using a glass burette to maintain the Ca + Zn/P molar ratio matching stoichiometric hydroxyapatite of 1.67. The pH value of the whole mixture was kept near 10 during the whole co-precipitation method by adding a few drops of ammonia through the continuous measuring of pH using a pH meter (Jenway 3510, England). After adding the whole amount of phosphate precursor, the white precipitate was continuously stirred at 80°C for 3 h and then left in its mother liquor for 2 days before vacuum filtration, washing, and drying in the oven at 100 ^o^C.

The functional groups present in ZnHA were ascertained *via* Fourier transform infrared spectroscopy (FT-IR) (Nicolet spectrometer model 670 with an FT-Raman accessory, United States). The crystalline phase of ZnHA nanoparticles was measured *via* an X-ray (XRD) device (BRUKER, D8 ADVANCE) with a monochromatic X-ray at a wavelength of 0.1542 nm using a Cu Kα source with a 200 mA emission current and a 40 kV voltage. The diffraction patterns were collected with an incremental step size of 0.02°C over 2 theta range of 5–60° with 2 s acquisition time for each scan. Transmission electron microscopy (TEM; JEOL JEM -2100) was used to monitor the particle size of Zn-doped hydroxyapatite with an accelerating voltage of 200 kV. The surface charge of ZnHA nanoparticles was characterized using Malvern Instruments Zetasizer Nano ZS90 (UK) after dispersing 250 μg/ml of the powder in 10 mM potassium chloride electrolyte solution.

Zinc ions content in HA dried powder was quantitatively measured using an inductively coupled plasma–optical emission spectrometer (Agilent Technologies 5100; ICP-OES) after digesting a certain weight of ZnHA powders in nitric acid.

### Experimental animals

In this study, 35 mature female Sprague–Dawley rats weighing (200–250) g were attained from the Animal Unit at the Faculty of Pharmacy, Mansoura University. The rats were acclimatized 2 weeks before the experiment in a controlled environment with 12-h light–dark cycles, a temperature of 22°C, and relative humidity of 65–70%. Throughout the experiment, the rats had unlimited access to standardized chow and water. The animals were cared for by following the Guide for the Care and Use of Laboratory Animals, which was authorized by the ethical committee of Mansoura University’s Faculty of Veterinary Medicine, with the registration number (R/94).

### Study design

**FIGURE 1:**
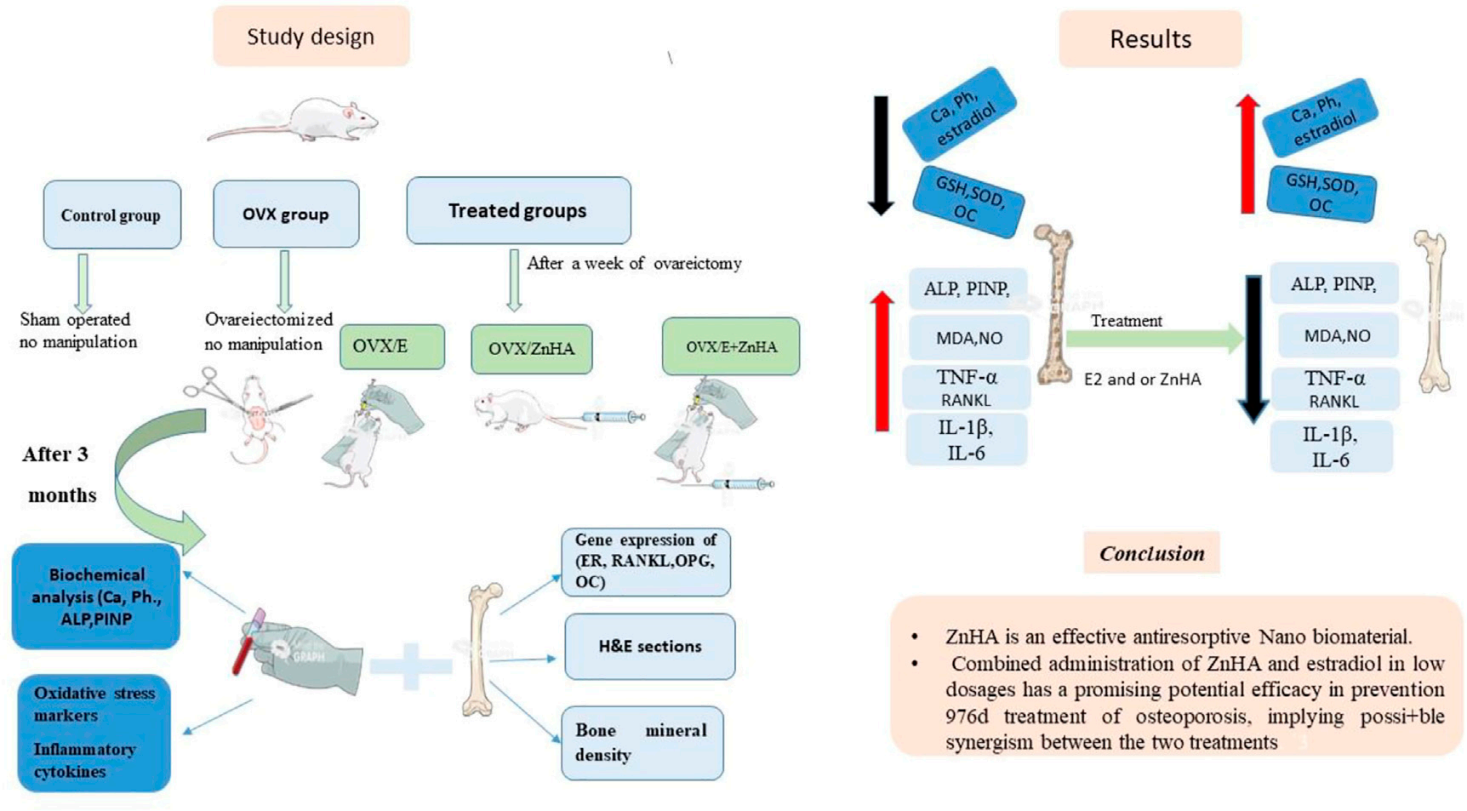
Summary of the study.

Animals were divided into five experimental groups (*n* = 7/group): 1) Control group (SHAM operated); 2) Ovariectomized (OVX) group; 3) OVX plus estradiol therapy (20 μg/kg (Zhang et al., 2019); OVX/E2) group; 4) OVX plus ZnHA-NPs therapy (500 μg/kg (Sahana et al., 2013) (OVX/ZnHA); and 5) a combination treatment of E2 (10 μg/kg plus ZnHA-NPs (250 μg/kg) (OVX/E2+ZnHA) group. Treatments with E2 and/or ZnHA started after a week following the surgery and continued through 90 days. Estradiol was orally administrated every second day (Zhang et al., 2019), while ZnHA was injected intravenously (i.v) twice during the experimental periods, first on Day 7 and second on Day 45 after ovariectomy (Sahana et al., 2013). The experimental design was summarized in Figure 1.

All surgical procedures of ovariectomy were performed under complete aseptic conditions, and a surgical plane of anesthesia was achieved using an intraperitoneal injection of 4 mg kg^−1^ xylazine HCL (Xylaject; Adwia, Egypt; 20 mg/ml) and 75 mg kg^−1^ ketamine (Ketalite 5%, ELICE PHARMA, Pakistan). To induce osteoporosis, bilateral ovariectomy was performed on each rat in all groups except the control group; rats in the control group were sham-operated under the same surgical procedure without excision of the ovaries. For ovariectomized rats, the two ovaries were exteriorized from the abdominal cavity through a 1 cm longitudinal midline incision, the mesovarium was ligated, and each ovary was removed, followed by the closure of the incision. Post-operative ketoprofen (Ketolgin^®^, AMOUN pharmaceutical Co., S.A.E.) at a dose of 3 mg/kg/day and penicillin G at a dose of 40,000 U/kg/day were injected intramuscular (I.M) for three successive days. To avoid possible contamination and cannibalism, the ovariectomized rats were housed individually for ten days, and later on, the sutures were removed**.**

### Sample collection

At the end of the experiment, blood samples were withdrawn from the medial canthus of the eye of each rat in the experimental groups and centrifuged for 10 min at 3000 r.p.m to separate clear serum samples that were carefully transferred to Eppendorf tubes to be stored at -20°C until used for further analysis. After euthanasia, the right femurs were removed rapidly and prepared for histological and gene expression analysis, whereas the left femurs were used to assess bone density.

### Analysis of serum biochemical parameters

Alkaline phosphatase (ALP) was estimated using commercial diagnostic kits (Teco diagnostics, California, United States) (catalog No.; A504-150). Moreover, serum estradiol (E2, catalog No.; MBS263466) and procollagen type I. N-terminal propeptide (P1NP, catalog No.; MBS007345) levels were determined following the manufacturer’s instructions of specific ELISA kits purchased from MyBioSource company (San Diego, United States). Calcium (Ca) was determined using the quantitative colorimetric Spinreact method described by Young (2001). Phosphorus (P) was determined according to Tietz (1990) by quantitative determination of inorganic phosphorous. All parameters were spectrophotometrically detected (5010 photometers, BM Co., Berlin, Germany) according to their enclosed pamphlets.

### Gene expression analysis

Reverse transcription-quantitative polymerase chain reaction (RT-qPCR) analysis was used to determine the expression of estrogen receptor (ER-α, ER-β), osteocalcin (OC), receptor activator of NF-kB ligand (RANKL), and osteoprotegerin (OPG) mRNAs in the femur bone. Glyceraldehyde-3-phosphate dehydrogenase (GAPDH) was used as the reference housekeeping gene to normalize the data. The RNA was extracted from the bone using TRIzol reagent (Invitrogen Life Technologies), and it was reverse-transcribed into cDNA using TOPscript™ RT DryMIX (Enzynomics Co. Ltd, Korea) according to the manufacturer’s protocol. qRT-PCR was utilized using Quanti Fast SYBR Green RT-PCR kit (Qiagen) to determine gene expression. Sense and antisense primers (Table1) were prepared by Eurofins Genomics (©Eurofins Genomics, Germany). qRT-PCR analyses were performed using ViiA™ seven System (Thermo Fisher Scientific) in triplicate. The fold-change was estimated using the ΔΔCt method (Livak and Schmittgen, 2001).

**TABLE 1:** PCR primers sequences of the studied genes.

Gene	*Accession number*	*Sense (5′-3′)*	*Antisense (5′-3′)*
ER-α	AY_280663.1	GCA​CAT​TCC​TTC​CTT​CCG​TC	GTA​GAG​AGC​TGA​GCA​CTA​GCT​T
ER-β	NM_012754.3	TAC​AGT​CCT​GCT​GTG​ATG​AAC​TAC​A	ACT​AGT​AAC​AGG​GCT​GGC​AC
OC	M_25490.1	CCTTTGGGT TTGACCTATT GCG	TCA​TGG​TGT​CTG​CTA​GGT​CTG
RANKL	NM_057149.1	CAC​AGC​GCT​TCT​CAG​GAG​TT	GAT​GGT​GAG​GTG​AGC​AAA​CG
OPG	U_94330.1	GCTGGCACACGAGTGATG	GCA​GAA​TTC​GAG​CTC​CAG​GTA
GAPDH	NM_017008.4	AGT​GCC​AGC​CTC​GTC​TCA​TA	TCC​CGT​TGA​TGA​CCA​GCT​TC

### Analysis of serum cytokines

Commercial ELISA kits from Quantikine Co. (United States) were used to determine concentrations of tumor necrosis factor-alpha (TNF-α, catalog No.; STA00D), interleukin-1β (IL-1β, catalog No.; MLB00C), and interleukin-6 (IL-6, catalog No.; S6050) in serum.

### Analysis of oxidative stress and antioxidant markers

The malondialdehyde (MDA, catalog No.; MD 25 29), nitric oxide (NO, catalog No.; NO 25 33), (NO), superoxide dismutase (SOD, catalog No.; SD 25 21), and reduced glutathione (GSH, catalog No.; GR 25 11) were determined in serum calorimetrically using Bio-diagnostic kits (Egypt) according to the manufacturers' guide.

### Histological and immunohistochemical analyses of the femur bone

Each femur bone from the experimental groups was dissected free of soft tissue and cleaned in saline. Samples were fixed in 10% neutral buffer formalin for 48 h before being decalcified in EDTA (10%) with exchanges of EDTA solution daily for 6 weeks (Drury and Wallington, 1980). The decalcified samples were cut longitudinally in a coronal plane along the central portion, dehydrated in ascending concentrations of ethyl alcohol, cleared in xylene, and embedded in liquid paraffin wax. Serial sections (5 μm thick) were obtained using a rotatory microtome for histological and immunohistochemical analyses. Sections were stained with hematoxylin and eosin (H&E) for general histological analyses and Masson Trichrome stain for assessing the density of collagen fibers, as described by Bancroft (2013).

The immunohistochemical protocol vascular endothelial growth factor (VEGF) and proliferating cell nuclear antigen (PCNA) was performed according to the method used by Petrosyan et al. (2002). In brief, 5 μm sections of bone were mounted on coated positive glass slide, dewaxed, rehydrated, incubated with 3% hydrogen peroxide for 15 min at room temperature to quench endogenous peroxidase, and blocked with 5% normal goat serum for 15 min. The sections were then incubated with specific antibodies against VEGF (polyclonal; rabbit; 1:50 dilution; Santa Cruz Biotechnology, Santa Cruz, CA) or PCNA (rabbit; monoclonal antibody; 1:100 dilution; GTX12496; Dako North America, Inc., Carpinteria, CA, United States) in a humidified dark chamber overnight at 4 °C. After that, the sections were washed with PBS and incubated with a biotin-conjugated secondary antibody (Cat. No. SC-2040) for 1 h, washed with PBS, and stained with 3,3′-diaminobenzidine before being counterstained with Mayer’s hematoxylin and examined using a light microscope (describe the microscope).

Histomorphometric analyses were carried out using the ImageJ analysis program (version 1.36, NIH, United States). Five rats from each group were selected randomly for morphometric analyses of their femur bone using three stained sections/rat (10 fields/section) for each group. The mean area of trabecular bone, the mean thickness of the outer cortical bone, and the mean number of osteocytes were determined (Zhou et al., 2015). Additionally, the mean percentage of collagen fiber density (Nasser et al., 2013) and the percentage of cells expressing VEGF and PCNA when analyzed at magnification were detected.

### Mineral density of femur bones

Computed topography (CT) was performed on femurs for the experimental groups using an orthodontic-grade cone-beam computed tomography (CBCT) scan (120 kV, 5 mA; i-CAT next generation; Imaging Sciences International, Hatfield, United States) at a clinically typical 0.125-mm voxel size and field of view dimensions of 16 cm diameter х 4 cm height. The mineral densities (BMD) of cortical dense (compact) bone in the mid-diaphyseal regions of the femur and the trabecular (cancellous) bone at the femur extremities were quantified using a quantitative CT method yielding Hounsfield units, as described by Chityala et al. (2004). Because BMD is not uniform in the same CT image, five-point densities were measured inside a specified 5 x 5 voxels diameter region of interest (ROI), and the mean ± SME was calculated.

### Data analysis

One-way analysis of variance, Duncan’s multiple comparison tests were used to detect statistical differences among all tested parameters using the SPSS 26.0 computer program. Data are presented as means ± standard errors of means (SME). *p*-values of 0.05 were considered to be statistically significant.

## Results

### Spectroscopic analyses of Zn-doped hydroxyapatite phase, (A) FT-IR spectrum, (B) XRD diffraction pattern.

**FIGURE 2:**
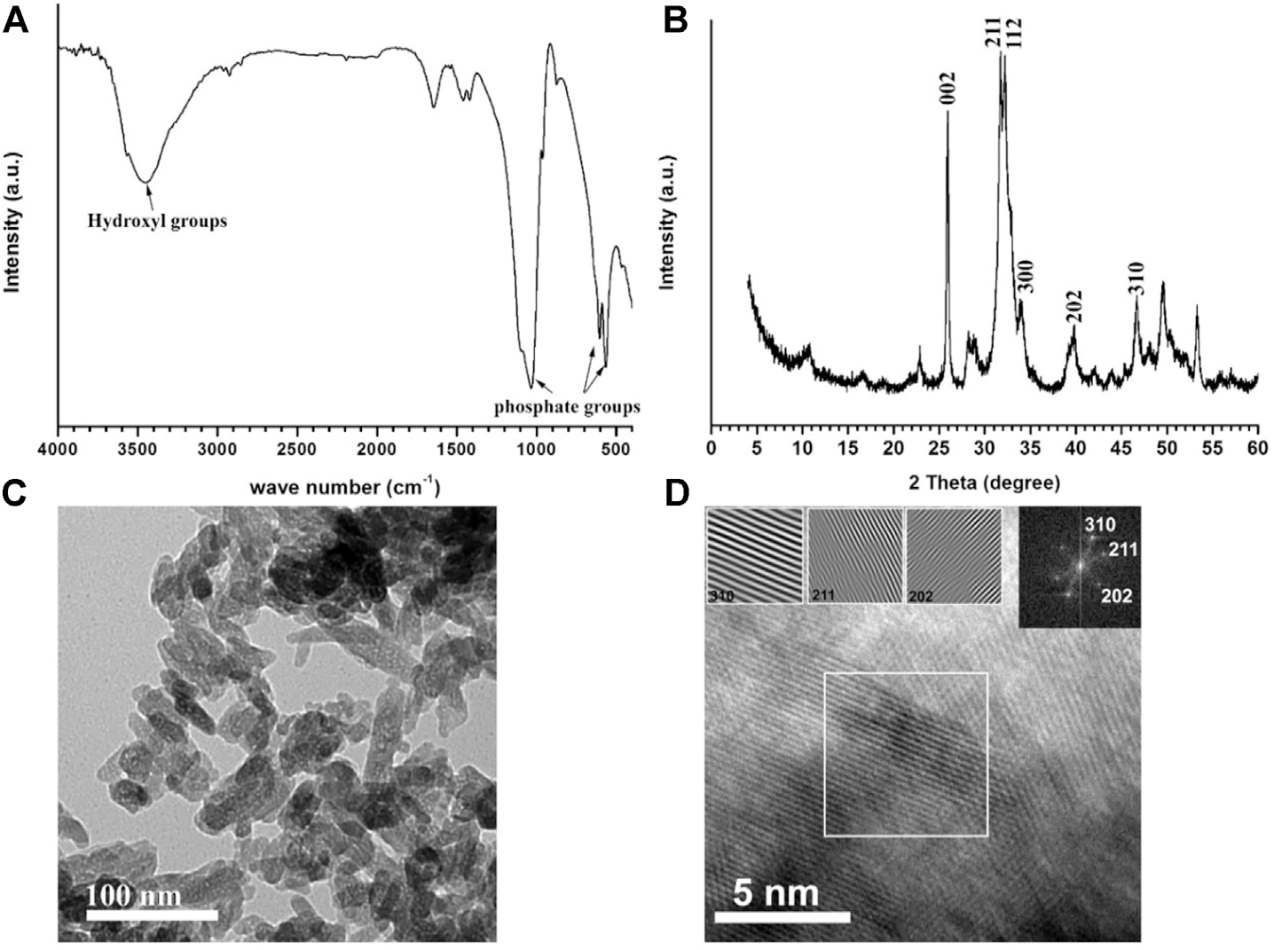
Spectroscopic analyses of Zn-doped hydroxyapatite phase, **(A)** FT-IR spectrum, and **(B)** XRD diffraction pattern. TEM micrograph **(C)** and HR-TEM **(D)** of Zn-doped hydroxyapatite. The inset image on the right-hand side in **(D)** represents the Fast Fourier Transform (FFT) pattern of HR-TEM, showing three hkl-reflections (310, 211, and 202) of HA phase and three images of simulated lattice fringes calculated from the area inside the white-bordered square in HR-TEM image.

Figure 2 represents the spectroscopic phase analyses of ZnHA-NPs prepared by the chemical precipitation method. Figure 2A confirms the presence of all typical vibrational bands of hydroxyapatite nanoparticles, including phosphate and structural lattice hydroxyl groups, which were located at 470, 550–600, 960, 1031, 1100 cm-1, and 3570 cm-1, respectively (Abdel-Fattah et al., 2008; Mardziah et al., 2021). Figure 2B shows all the diffraction peaks of the XRD pattern that are matched with the diffraction peaks of stoichiometric hydroxyapatite of ICDD (JCPDS) standards card # 9–432. The crystallite size of the 002 planes along the c-axis of hydroxyapatite hexagonal crystal was calculated using the Scherrer equation and was found to be close to 30 nm. All miller indices (hkl) are presented in Figure 2B, which represents the most intense peak of the crystal structure of hydroxyapatite, indicating the successful formation of the hydroxyapatite phase.

Figure 2 also shows TEM micrograph (C) and HR-TEM (D) of Zn-doped hydroxyapatite; the inset image on the right-hand side in Figure 2D represents the fast Fourier transform (FFT) pattern of HR-TEM, showing three hkl-reflections (310, 211, and 202) of HA phase and three images of simulated lattice fringes calculated from the area inside the white-bordered square in HR-TEM image. The particle size and shape were inspected using the TEM technique at a high voltage (Figure 2), indicating that all the particles have a rod-like shape with an aspect ratio (average ratio of length and width) of 3.18 ± 0.77. The average length of the 100 particles measured *via* Nano measure (version 1.2.5) was found to be equal to 64 ± 10 nm, and the average width was equal to 20 ± 3 nm. The zeta potential of nanoparticles was equal to -19.4 ± 3.94 mV, which is consistent with previous work (2017 Predoi Materials)*.*

The zinc content measured using the ICP-OES technique was found to be 600 ± 11.7 mg per kilogram of ZnHA powder. This result is much higher than the ones previously reported as a contaminant in biogenic and commercial synthetic hydroxyapatite powders (Giraldo-Betancur et al., 2013), confirming the successful incorporation of Zn ions in HA lattice with a much higher amount.

### The effects of E2 and ZnHA-NPs treatments on the bone turnover markers and estrogen level in ovariectomized (OVX) rats

As shown in Table 2, the levels of serum ALP (*p* = 0.0001) and PINP (*p* = 0.0001) in OVX rats were markedly elevated with lowered calcium (*p* = 0.0001), phosphorus (*p* = 0.0002), and estradiol (*p* = 0.0001) levels, when compared with the control group. However, significant improvement in ALP (*p* = 0.0001) activity, PINP (*p* less than 0.0001), calcium (*p* less than 0.0001), phosphorus (*p* = 0.0008, 0.0001, less than 0.0001), and estradiol (*p* = 0.0001) levels toward normal level was observed in all experimental groups (OVX/E, (OVX/ZnHA, OVX/E + ZnHA), especially in the combined treated group (OVX/E + ZnHA) when compared to the OVX model group (*p* < 0.05).

**TABLE 2:** Effects of estrogen (E2) and zinc hydroxyapatite (ZnHA) treatments on concentrations of serum bone turnover markers of control and treated rats.

Parameters	Experimental groups				
	Control	OVX	OVX/E	OVX/ZnHA	OVX/E + ZnHA
ALP (U/L)	385.56 ± 12.71^**e**^	913.09 ± 24.28^**a**^	720.37 ± 12.30^**b**^	635.11 ± 7.67^**c**^	564.34 ± 18.37^**d**^
PINP (ng/ml)	5.11 ± 0.42^**e**^	9.96 ± 0.26^**a**^	8.35 ± 0.09^**b**^	7.28 ± 0.25^**c**^	6.27 ± 0.04^**d**^
Calcium (mg/dl)	11.98 ± 0.21^**a**^	6.83 ± 0.22^**e**^	9.20 ± 0.08^**d**^	10.94 ± 0.38^**c**^	12.81 ± 0.19^**b**^
Phosphorus (mg/dl)	6.69 ± 0.44^**a**^	3.88 ± 0.17^**c**^	5.15 ± 0.09^**b**^	5.69 ± 0.16^**b**^	6.24 ± 0.14^**ab**^
Estradiol E2 (pg/ml)	16.22 ± 1.58^**a**^	4.89 ± 0.29^**d**^	11.44 ± 0.39^**b**^	7.22 ± 0.06^**c**^	9.21 ± 0.17^**c**^

Data were expressed as mean ± SME (*n* = 5/group, triplicate). Means in the same raw with different superscripts are significantly different (*p* < 0.05). ALP, alkaline phosphatase; PINP, procollagen type I N-terminal propeptide.

a Means in the same raw with different superscripts are significantly different (*p* < 0.05).

b Means in the same raw with different superscripts are significantly different (*p* < 0.05).

c Means in the same raw with different superscripts are significantly different (*p* < 0.05).

d Means in the same raw with different superscripts are significantly different (*p* < 0.05).

### The effects of E2 and ZnHA-NPs treatments on femoral target genes in ovariectomized rats

**FIGURE 3:**
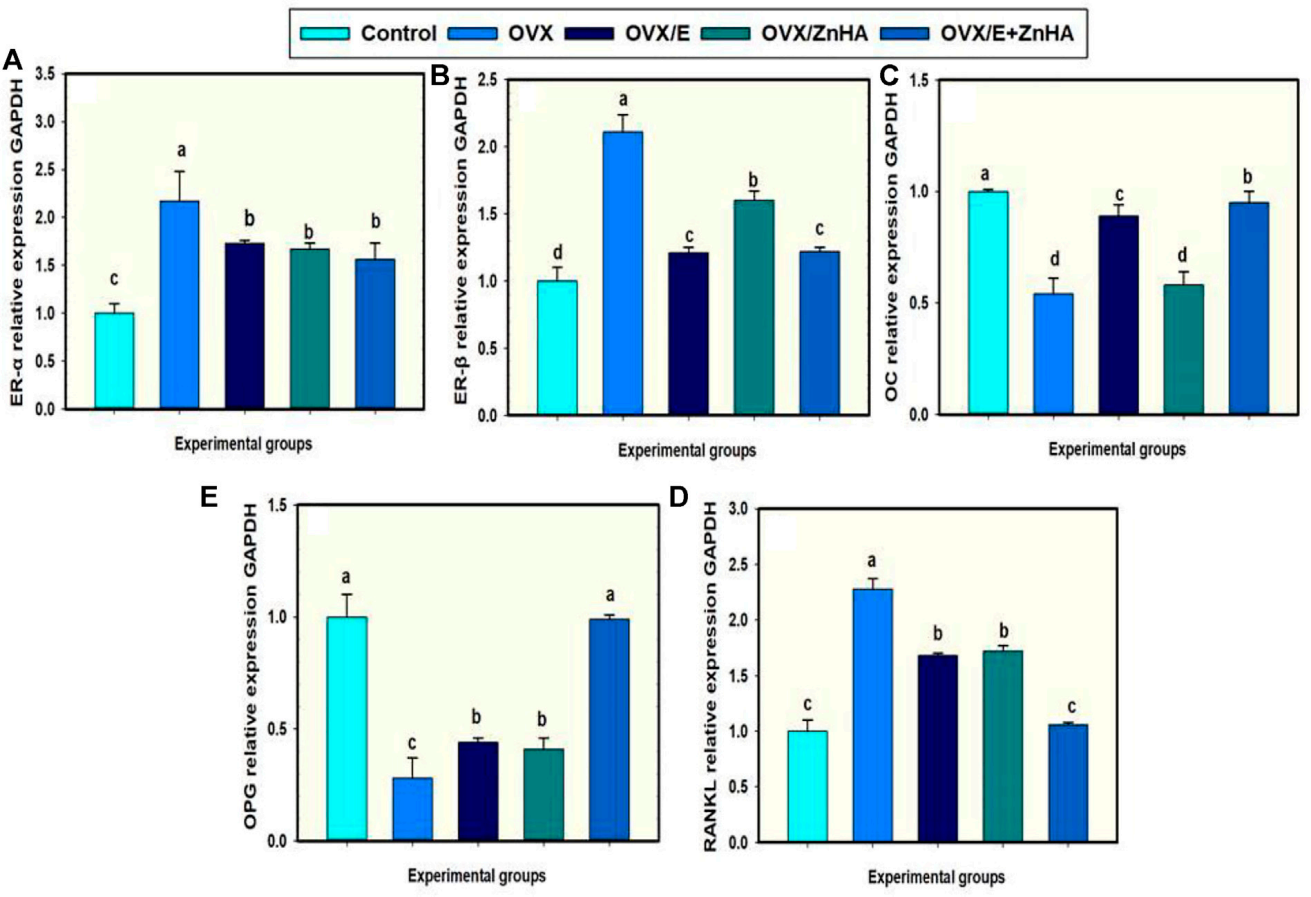
Effects of estrogen (E2) and/or zinc hydroxyapatite nanoparticles (ZnHA-NPs) on mRNA expression of femoral target genes in ovariectomized (OVX) rats. Data were expressed as mean ± SME (*n* = 5/group, triplicate). Bars carrying different superscripts are significantly different (*p* < 0.05). ER-α, Estrogen receptor alpha **(A)**; ER-β, Estrogen receptor beta **(B)**; OC, Osteocalcin **(C)**; RANKL, receptor activator of nuclear factor-kappa B (NF-kB) ligand **(D)**; OPG, osteoprtogrin **(E)**.

Data in Figure 3 reveal that femoral expression of ER-α (*p* = 0.0010), ER-β (*p* = 0.0049), and RANKL (*p* = 0.0091) mRNAs of the OVX group were upregulated when compared to the control group associated with the significant decline in the expression of OPG (*p* = 0.0091) and OC (*p* = 0.0055) mRNAs. However, administration of E2 and/or ZnHA resulted in a decrease in the expression of ER-α (*p* = 0.0016, 0.0379, 0.0052), ER-β (*p* = 0.0149, 0.0372, 0.0044), and RANKL (*p* = 0.0099, 0.0305, 0.0012) and elevation in the expression of OPG (*p* = 0.0116, 0.0305, 0.0012) and OC (*p* = 0.0036, 0.6543, 0.0014) in femur when compared to OVX group. The combined treatment in group OVX/E2+ZnHA was more effective than using each treatment alone and returning to normal levels of RANKL and OPG mRNAs (*p* < 0.05).

### The effects of E2 and ZnHA-NPs treatments on the serum cytokines levels in ovariectomized rats

**FIGURE 4:**
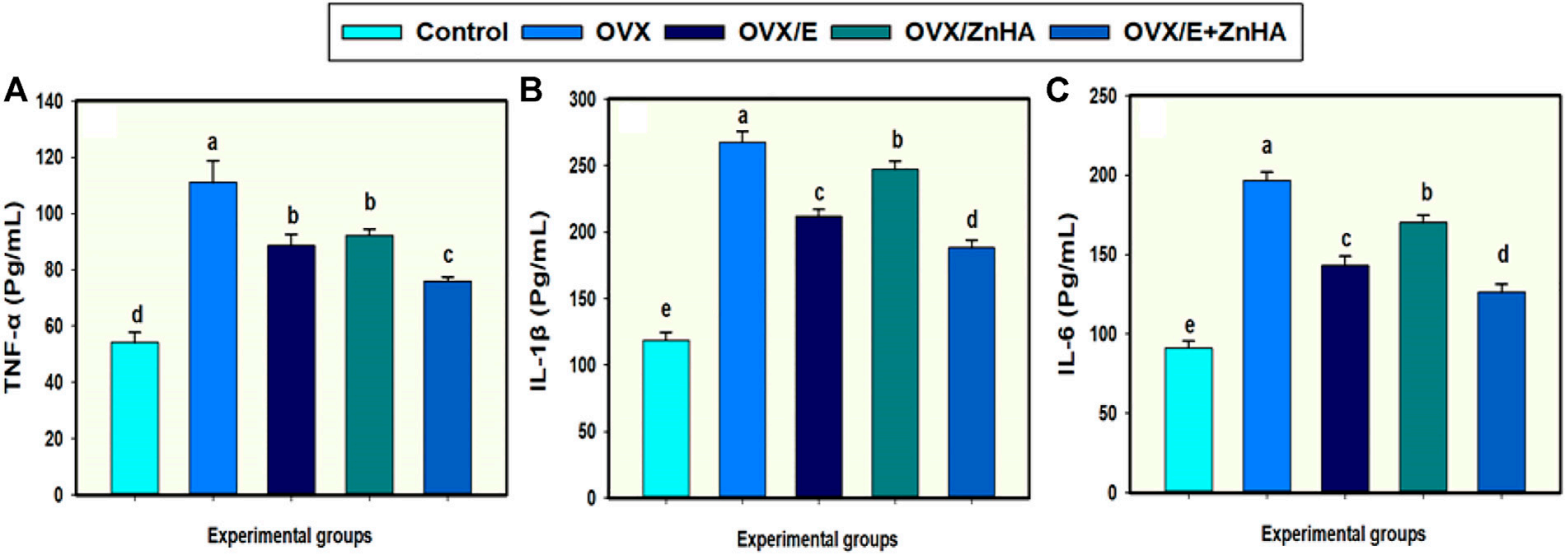
Effects of estrogen (E2) and/or zinc hydroxyapatite nanoparticles (ZnHA-NPs) on serum concentrations of cytokines in ovariectomized (OVX) rats. Data were expressed as mean ± SME (*n* = 5/group, triplicate). Bars carrying different superscripts are significantly different (*p* < 0.05). TNF-α, tumor necrosis factor-alpha **(A)**; IL1B, Interleukin-1β **(B)**; IL-6, Interleukin-6 **(C)**.

Data in Figure 4 show that ovariectomy (OVX group) significantly elevated the levels of serum TNF-α (*p* = 0.003), IL-1β (*p* = 0.0001), and IL-6 (*p* = 0.0001) as compared to the control rat. Treating OVX-rats with E2 and/or ZnHA-NPs significantly reestablished the serum TNF-α (*p* = 0.033, 0.0417, 0.0021), IL-1β (*p* = 0.005, 0.0378, less than 0.0001), and IL-6 (*p* = 0.002, 0.018, less than 0.0001) relative to those for untreated OVX rats. The OVX-rats treated with E2 and ZnHA-NPs (OVX/E + ZnHA) had a lower inflammatory response when compared with the OVX model group (*p* < 0.05).

### The effects of E2 and ZnHA-NPs treatments on the serum oxidative/antioxidant biomarkers in ovariectomized rats

Regarding the oxidative stress markers (Table 3), higher levels of serum MDA (*p* = 0.031) and NO (*p* = 0.0001) were detected in OVX rats associated with lower SOD (*p* = 0.017) activity and GSH (*p* = 0.006) level when compared to the control rats. The co-administration of E2 alone or with ZnHA-NPs restored the NO (*p* = 0.0015, 0.0275, 0.0430) level relative to the ovariectomized (OVX) group. The potential SOD (*p* = 0.0051, 0.4557, 0.0510) activity and GSH (*p* = 0.0159, 0.5140, 0.0888) level were increased only in the OVX/E group parallel to that of the OVX group.

**TABLE 3:** Effects of estrogen (E2) and zinc hydroxyapatite nanoparticles (ZnHA) treatments on serum oxidative/antioxidant biomarkers of control and treated rats.

Parameters	Experimental groups				
	Control	OVX	OVX/E	OVX/ZnHA	OVX/E + ZnHA
MDA (nmol/L)	6.98 ± 0.80^**b**^	11.90 ± 1.39^**a**^	9.78 ± 0.42^**a**^	11.88 ± 0.49^**a**^	10.61 ± 0.54^**a**^
NO (µmol/L)	12.22 ± 0.51^**c**^	33.37 ± 1.41^**a**^	27.46 ± 0.42^**b**^	29.61 ± 0.52^**ab**^	28.17 ± 0.91^**b**^
SOD (U/L)	107.67 ± 9.97^**a**^	63.73 ± 4.85^**c**^	84.89 ± 2.70^**b**^	59.09 ± 4.25^**c**^	77.32 ± 4.37^**bc**^
GSH (mg/dl)	4.97 ± 0.36^**a**^	1.99 ± 0.43^**c**^	3.50 ± 0.24^**b**^	2.31 ± 0.09^**c**^	2.77 ± 0.32^**bc**^

Data were expressed as mean ± SME (n = 5/group, triplicate). Means in the same raw with different superscripts are significantly different (*p* < 0.05). MDA, malondialdehyde; NO, nitric oxide; GSH, reduced glutathione; SOD, superoxide dismutase.

a Means in the same raw with different superscripts are significantly different (*p* < 0.05).

b Means in the same raw with different superscripts are significantly different (*p* < 0.05).

c Means in the same raw with different superscripts are significantly different (*p* < 0.05).

d Means in the same raw with different superscripts are significantly different (*p* < 0.05).

### The effects of E2 and ZnHA-NPs treatments on the femoral histological picture and immunohistochemical expression of VEGF and PCNA in ovariectomized rats

**FIGURE 5:**
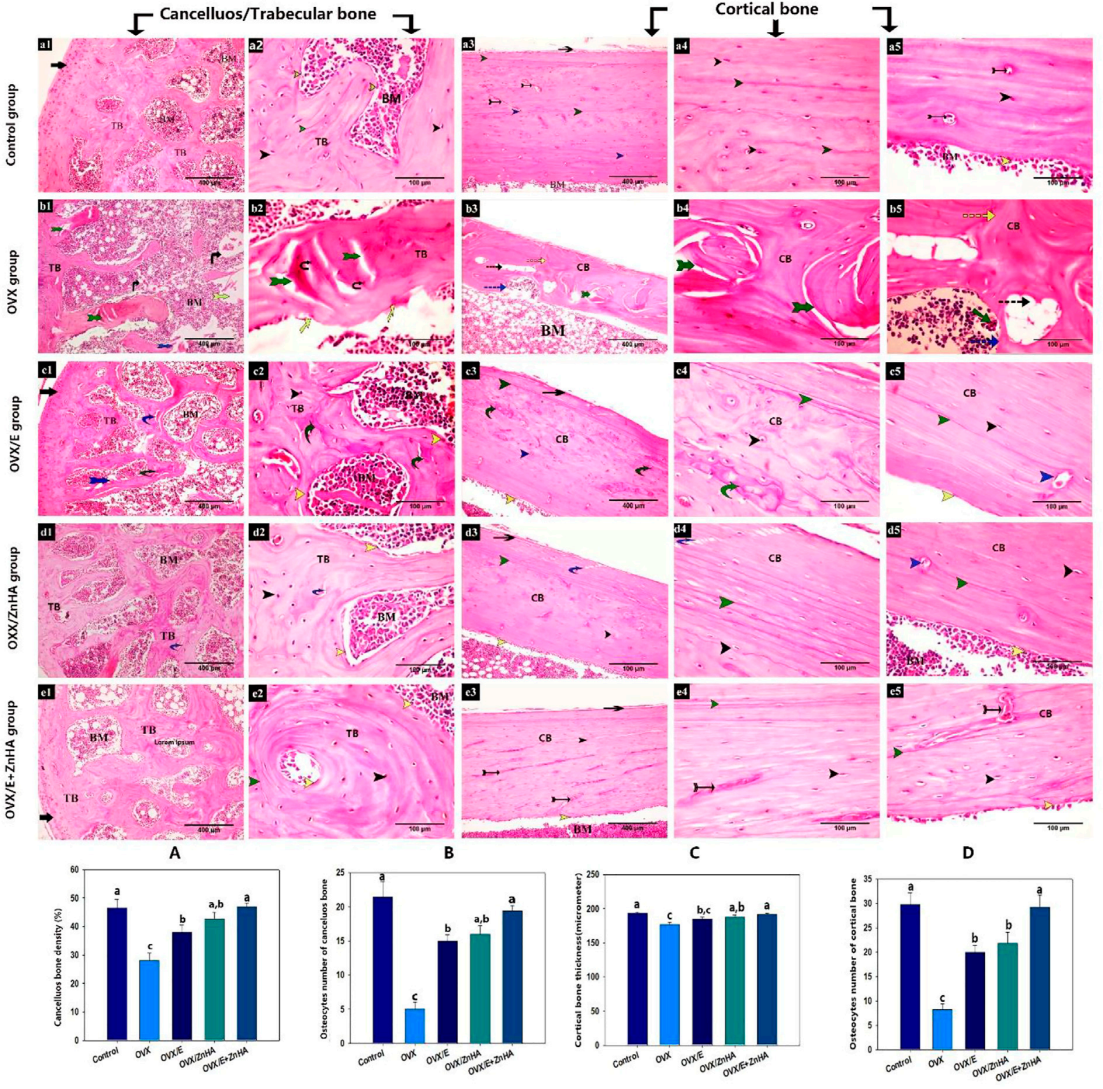
Photomicrograph of the bone tissue stained with H & E following estrogen (E2) and/or zinc hydroxyapatite nanoparticles (ZnHA-NPs) in ovariectomized (OVX) rats showing: articular cartilage (black arrow), anastomosing and thick bone trabeculae (TB), bone marrow cavity (BM), cortical bone (CB), lacunae containing osteocytes (black arrow head), distinct cement lines (green arrow head), smooth, continuous endosteal surface with osteogenic and osteoblast cells(yellow arrow head), Haversian canal (blue arrow head), blood vessel (tailed black arrow), overlying periosteum (blue arrow), thin bone trabeculae (tTB), distorted and cracked bone trabeculae (tailed green arrow), necrotic osteocytes (black tailed arrow), bone spicule island(s), distorted area of bone marrow (yellow tailed arrow), eroded endosteal surface with osteoblast absence (yellow arrow), cortical bone with disorganized bone matrix (discontinues green arrow) or with many resorption vacuoles (discontinuous black arrow), how ship’s (discontinues blue arrow) containing osteoclasts (green arrow). Note that some cracks (curved blue arrow) were detected in bones from OVX/E2 and OVX/ZnHA rats or a mosaic bony matrix appearance (curved green arrow) and discontinuous cement lines (red arrow head) were detected in bone from OVX/E2 rats. (a1,2) trabecular bone (a3-5) cortical bone of control group. (b1,2) trabecularbone (b3-5) cortical bone of OVX group. (c1,2) trabecular bone (c3-5) cortical bone of OVX/E group. (d1,2) trabecular bone (d3-5) cortical bone of OVX/ZnHA group. (e1,2) trabecular bone (e3-5) cortical bone of OVX/E+ZnHA group. Histomorphometric analysis **(A-D)** were expressed as mean ± SME (*n* = 5/group, triplicate). Bars carrying different superscripts are significantly different (*p* < 0.05).

As shown in Figure 5, the classical appearance of cancellous/trabecular and cortical bone was detected. The osteocytes were distributed within the bone matrix inside their lacunae, and the endosteal surface appeared smooth and lined with osteoprogenitor and osteoblast cells. In contrast, the examination of stained sections from the OVX group revealed an evident histological alteration in the form of distortion and appearance of many resorption vacuoles associated with a significant decrease in the mean area of the trabecular bone density (*p* = 0.002) and cortical bone thickness (*p* = 0.0014) compared to the control group. Furthermore, the endosteal surface appeared eroded with the absence of osteogenic and osteoblast lining and the appearance of osteoclast within the how ship’s lacuna. The osteocyte numbers were significantly decreased in the trabecular (*p* = 0.0002) and cortical bones (*p* less than 0.0001) compared to the control group.

The obvious improvement in the histological architecture was manifested by a significant increase in the mean area of trabecular bone density and cortical bone thickness in OVX/E (*p* = 0.0317, non-significant in cortical shaft thickness), OVX/ZnHA (*p* = 0.0051 and 0.0403, respectively) and OVX/E + ZnHA groups (*p* = 0.0002 and 0.0029, respectively) compared to OVX group. In addition, the number of osteocytes was significantly increased in the trabecular and cortical bone of OXV/E (*p* less than 0.0001, = 0.0002, respectively), OXV/ZnHA (*p* = 0.0001 and 0.0008, respectively), and OVX/E + ZnHA (*p* less than 0.0001) groups compared to the OVX group.

### Masson Trichrome stain

**FIGURE 6:**
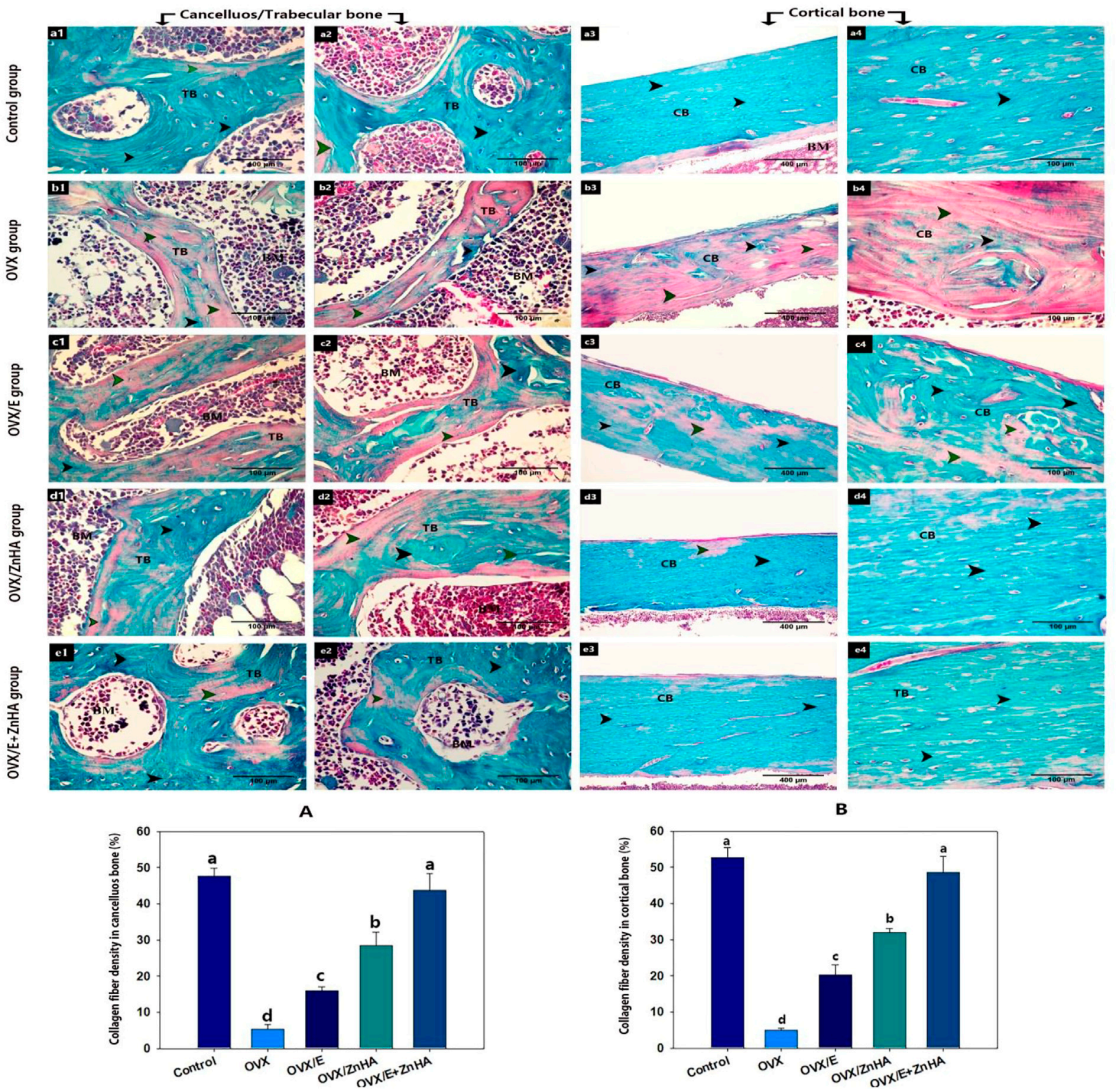
Photomicrograph of bone tissue stained with Masson Trichrome stain following estrogen (E2) and/or zinc hydroxyapatite nanoparticles (ZnHA-NPs) in ovariectomized (OVX) rats showing: matrix of the trabecular bone (TB) or cortical bone (CB) with green colored collagen fibers (black arrowhead) or without green colored collagen fiber (green arrowhead). (a1,2) trabecular bone and (a3,4) cortical bone of control group. (b1,2) trabecular bone and (b3,4) cortical bone of OVX group. (c1,2) trabecular bone and (c3,4) cortical bone of OVX/E group. (d1,2) trabecular bone and (d3,4) cortical bone of OVX/ZnHA group. (e1,2) trabecular bone and (e3,4) cortical bone of OVX/E+ZnHA group. Note the green colored area was widely distributed in control group and OVX/E+ZnHA followed by OVX/ZnHA group then OVX/E group. Histomorphometric analysis **(A,B)** were expressed as mean ± SME (*n* = 5/group, triplicate). Bars carrying different superscripts are significantly different (*p* < 0.05).

As presented in Figure 6, the trabecular and cortical bone of the control group exhibited that the bone matrix was mostly composed of green-stained collagen fibers, and the osteocytes were present inside their lacunae. The mean % of the green-colored area was significantly decreased (P is less than 0.0001) in the OVX group compared to the control group, while it significantly increased after estrogen (*p* = 0.0008) or ZnHA nanoparticles (*p* = 0.0013) administrations compared to the OVX group, while returning to the normal one in the OVX/E + ZnHA group.

### Immune expression of VEGF and PCNA

**FIGURE 7:**
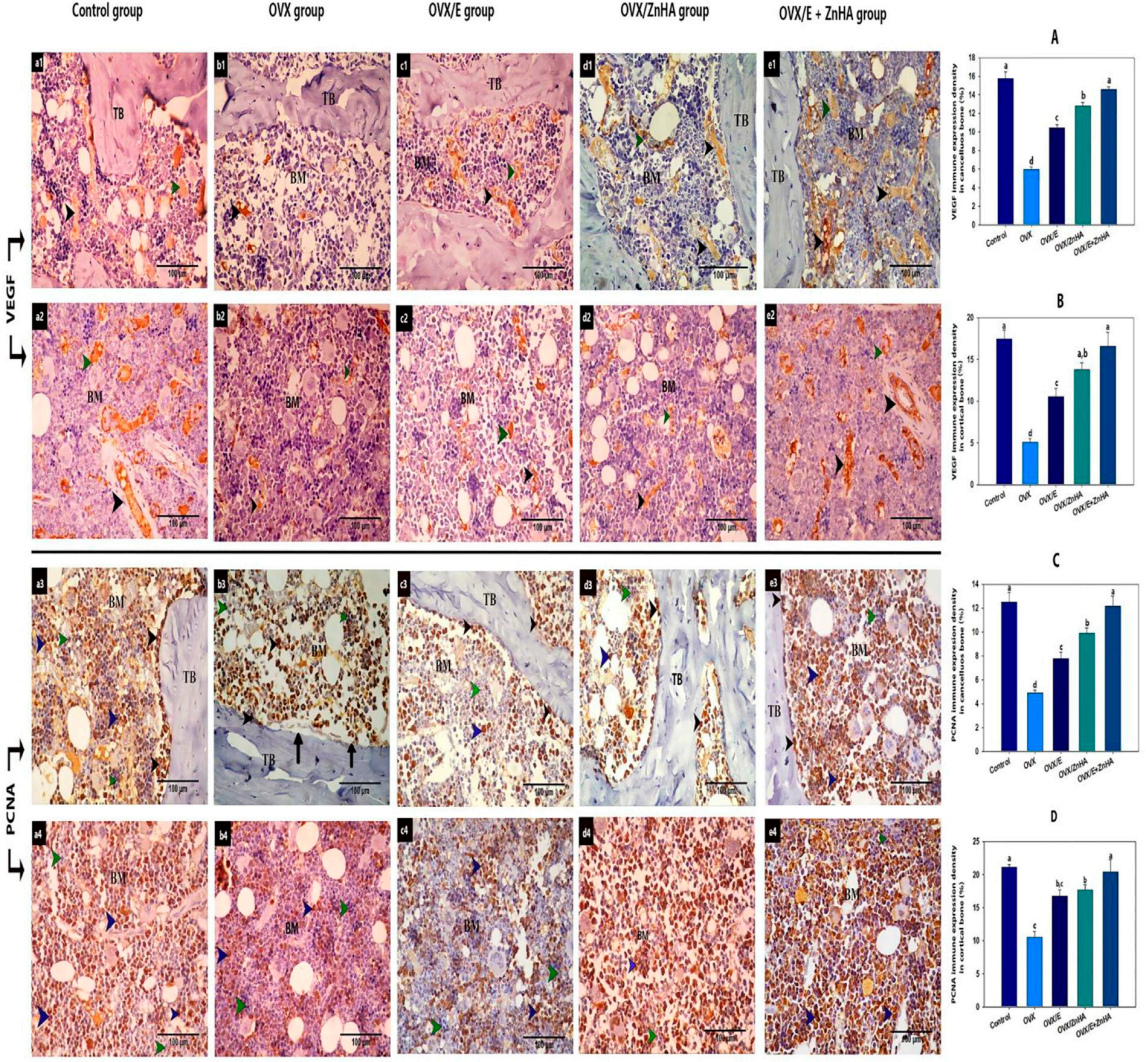
Immunohistochemical analyses of bone tissues for VEGF protein (a1,2- b1,2- c1,2- d1,2 and e1,2- and PCNA (a3,4-b3,4-c3,4- d3,4 and e3,4-) following estrogen (E2) and/or zinc hydroxyapatite nanoparticles (ZnHA-NPs) in ovariectomized (OVX) rats. VEGF was detected in blood vessels of bone marrow (black arrow head) and some hematopoietic cells (green arrow head). PCNA expression was detected in osteoblasts or osteogenic cells (black arrow head) and some cells in bone marrow (blue arrow head). Note that there are some PCNA-negative bone marrow cells (green arrow head). (a1,3-b1,3-c1,3-d1,3- and e1,3) trabecular bone from control, OVX, OVX/E, OVX/ZnHA and OVX/E+ZnHA groups respectively. (a2,4-b2,4-c2,4-d2,4 and e2,4) cortical bone marrow from control, OVX, OVX/E, OVX/ZnHA and OVX/E+ZnHA groups, respectively. Histomorphometric analysis **(A-D)** were expressed as mean ± SME (*n* = 5/group, triplicate). Bars carrying different superscripts are significantly different (*p* < 0.05).

As demonstrated in Figure 7, the expression of VEGF was mostly detected in blood vessels and some of the hematopoietic cells of the bone marrow of trabecular and cortical bones in the control group. VEGF expression was significantly decreased (*p* = 0.0003 and 0.0004, respectively) in the OVX group compared to the control group, but significantly increased in OVX/E (*p* = 0.0005 and 0.0067, respectively), OVX/ZnHA (*p* = 0.0002 and 0.0008, respectively), and OVX/E + ZnHA (P is less than 0.0001, = 0.0027, respectively) groups compared to the OVX group. The highest expression of VEGF was detected in the combined treatment (OVX/E + ZnHA).

The PCNA expression was detected in the osteoblast and osteogenic cells of the endosteum and some of the bone marrow cells in the control group. Few osteoblasts or bone marrow cells with positive PCNA expression were detected in the OVX group, where the mean % of PCNA expression for trabecular and cortical bone of this group was significantly decreased (*p* = 0.0070 and 0.0116, respectively) compared to the control group. A noticeably large number of osteoblast and bone marrow cells with positive PCNA expression was detected associated with a significant increase in PCNA expression in OVX/E (*p* = 0.0083 and 0.0073, respectively) OVX/ZnHA (*p* = 0.0006 and 0.0034, respectively), and OVX/E + ZnHA groups (*p* = 0.0001 and 0.0087, respectively) compared to the OVX group.

**FIGURE 8:**
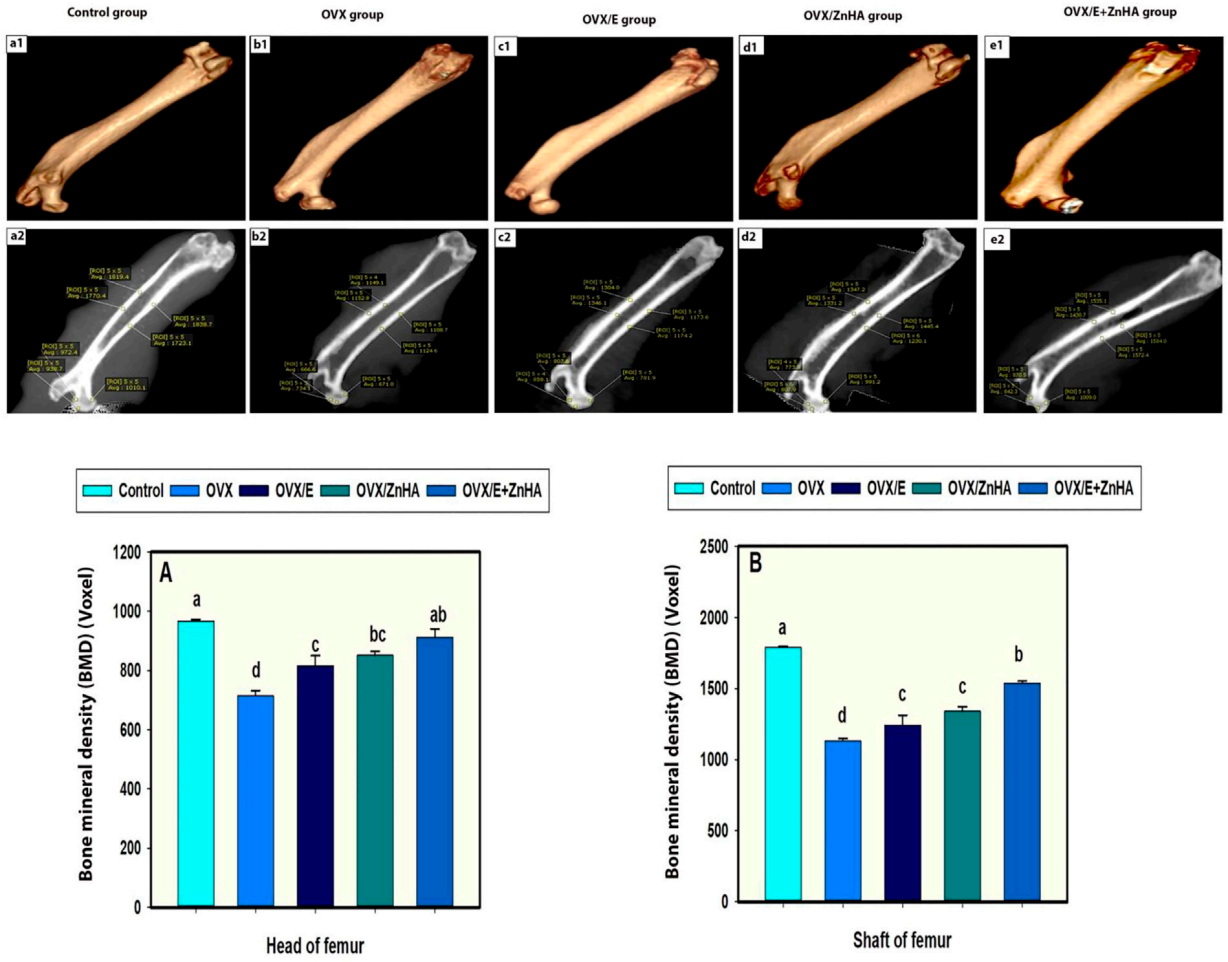
a1, b1, c1, d1 and e1 represent three-dimension image of femur within animal group. a2, b2, c2, d2 and e2 representing Average of bone mineral densities of both shafts and heads of femurs. (BMDs were measured in a region of interest [ROI) 5 x 5 voxels. Data of BMDs in head of femur **(A)** and shaft **(B)** expressed as Mean ± SEM. The different letters indicate significant differences (*p* < 0.05) among between experimental groups.

Results of the cone beam computed tomography (CBCT) analyses (Figure 8F) showed that the mineral density of the trabecular and cortical bone in the OVX group was lower (*p* = 0.0001) than in the control group. Administration of either E2 or ZnHA resulted in a slight improvement (*p* = 0.034 and 0.003, respectively) in the cortical BMD and induced (*p* = 0.029 and 0.002, respectively) moderate improvement in the trabecular BMD compared to that of the OVX group. Combined treatment in group OVX/E2+ZnHA had effectively alleviated (*p* = 0.0001) both cortical and trabecular BMD, whereas the cortical BMD was non-significant (*p* = 0.431) in the control group. The three-dimension image of the femur within the animal group is represented in Figure 8 (a1–e1).

## Discussion

Osteoporosis (OP) is a systemic skeletal disease characterized by loss of bone mass, deterioration of bone micro-architectonics, and an increase in the risk of fracture (Liu et al., 2015; Owen and Reilly, 2018). The current treatments for OP have notable limitations, including adequacy and long-term safety concerns (Khajuria et al., 2011). Until recently, there has not been good satisfactory therapy for OP (Arcos et al., 2014). Although HRT has a clear effect on the treatment and prevention of OP, estrogen therapy is not recommended for elderly women since the hazards outweigh the benefits (Arcos et al., 2014). As a result, there is an emergent need to discover more precise novel drugs with fewer side effects (Fouad-Elhady et al., 2020b). The current investigation is a pre-clinical trial for alternative potent and effective osteoporosis medication that can be administrated alone or with E2 in low dosages. Because of its similarity to bone minerals, hydroxyapatite has been a biocompatible ceramic in a variety of medical applications, primarily as bone substitutes (Fouad-Elhady et al., 2020a). HA is bioactive, osteoconductive, and capable of prompting the proliferation and attachment of bone cells and causing stem cells to differentiate into osteoblasts (Liang et al., 2021). Therefore, ZnHA-NPs can be considered a promising therapy in bone illness and osteoporosis.

Alkaline phosphatase is an important enzyme that is produced during the bone formation cycle and is therefore considered an excellent indicator of bone formation activity (Shidara and Inaba, 2009; Lowe et al., 2017) and BMD (Yang et al., 2021). In addition, procollagen type 1 N-terminal propeptide (P1NP) has been proven to be a more sensitive bone biomarker to determine the bone formation rate in osteoporosis (Kuo and Chen, 2017). Our findings revealed a significant elevation in serum ALP activity and PINP levels in the OVX group, which may elicit bone loss in this group, as higher levels of ALP were related to an imbalance between osteoblast and osteoclast, leading to an increase in osteoclast activity (Said et al., 2020; Tariq et al., 2021). In addition, serum ALP levels in osteoporotic rats significantly increased by 48.07% (Fouad-Elhady et al., 2020b). Moreover, the study by Garnero (2008) revealed that the mean value of total P1NP before initiating therapy was higher (74%) than that in healthy premenopausal women.

Calcium (Ca) and phosphorus (P) are essential components of the inorganic matrix of bone and are important contributors to bone health maintenance (Allan et al., 2008), and their deficiency causes defective bone mineralization, bone pathology, and clinical disease (Garnero and therapy, 2008). In comparison to other groups, bilateral ovariectomy resulted in a significant decrease in serum Ca, P, and estradiol levels in the OVX group, which correlated with previous results (Labah, 2017) (Prakash and Behari, 2008). This considerable drop in serum calcium levels suggested that ovariectomy led to the deterioration of bone mineralization (Liu et al., 2021). The decline in Ca levels may be related to dropping in serum E2 levels as a consequence of ovariectomy. This drop in E2 level changed the expression of numerous proteins involved in the re-absorption of Ca in distal renal tubules resulting in increased renal excretion of Ca (Sheweita and Khoshhal, 2007).

OC is the most abundant non-collagenous protein in bone produced by osteoblasts (Licini et al., 2019). It plays an important role in calcium homeostasis and mineralization of bone (Wang et al., 2021) and is, thus, closely linked to BMD. In the current study, rats in the OVX group had lower levels of OC mRNA expression in the femur bone than control rats. The OC is a calcium-dependent biomarker with a great affinity for the bone matrix responsible for mineralization (Jagtap and Bapat, 2013), thus, the lack of Ca and decreased mineralization in this group is the main cause of reducing femoral expression of OC.

Estrogen acts through two receptors [estrogen receptor-alpha (ER-α) and -beta (ER-β)] that are extensively expressed in bone cells and bone marrow (Ishimoto and Jaffe, 2011). ER-α is more important than ER-β for the regulation of bone metabolism (Streicher et al., 2017). Our results cleared that the OVX group had higher levels of mRNA expression of ER-α and ER-β, which may be correlated to the hypoestrogenism in this group. ER-β expression rises with age and is associated with the production of many pro-inflammatory markers and a decrease in VEGF and angiogenesis (Novella et al., 2012).

Cytokines are produced by activated monocytes/macrophages at the site of inflammation (Osama et al., 2014). TNF-α, IL-1β, and IL-6 are important regulators of bone cell number and activity, consequently affecting the rate and balance of bone turnover and controlling osteoclast development (Muhl and Pfeilschifter, 2003). The primary action of TNF-α and IL-1β is to activate mature osteoclasts and accelerate bone resorption *in vitro* and *in vivo* by stimulating the proliferation and differentiation of osteoblast precursors (Kitazawa et al., 1994). On the other hand, IL-6 prompts the development of osteoclasts from hemopoietic precursors (Nava-Valdivia et al., 2021). Additionally, bone resorption could be determined by the balance between these cytokines, RANKL, OPG, and bone mineral density (Recker et al., 2015). Our findings revealed a severe elevation in serum cytokines levels (TNF-α, IL-1β, and IL-6) in osteoporotic rats (OVX group), which may be associated with depletion of endogenous estrogen levels which induce an increment of apoptosis of osteocytes (Tomkinson et al., 1997). In addition, as significant upregulation of mRNA expression of RANKL in the femur, whereas OPG gene expression was downregulated in the OVX group. Our findings were previously reported in other published studies (Li et al., 2015; He et al., 2017; Fouad-Elhady et al., 2020b; Said et al., 2020). These results were confirmed by the appearance of osteoclast within the how ship’s lacuna and erosion in the endosteal surface with the absence of osteogenic and osteoblast lining as recorded in our histological screening.

The activity of osteoblast and osteoclast is disrupted by oxidative stress (Domazetovic et al., 2017). Reactive oxygen species (ROS) induce apoptosis of osteoblasts and osteocytes and the process of mineralization and osteogenesis slowdown (Bonewald, 2011). Glutathione (GSH) is the most crucial antioxidant in cells that represents the major defense tool *via* its chelating capacity (Elghareeb et al., 2021; Rezk et al., 2022). GSH is expressed by osteoclasts and prevents its production by the degradation of hydrogen peroxide (Lean et al., 2003). Additionally, nitric oxide (NO) is a signaling molecule that has been linked to bone function (Kalyanaraman et al., 2018), and the possible action of estrogen on bone appears mediated through NO (O'Shaughnessy et al., 2000). Inconsistently, the crucial role of SOD activity in preventing bone mass loss was confirmed in transgenic mice lacking cytoplasmic SOD (Smietana et al., 2010). In the current study, the levels of MDA and NO in the OVX group were significantly higher, whereas activities of antioxidants markers, GSH and SOD, were lowered than those for control rats, likely due to the decrease in E2 in OVX rats. Similar to other reports, reported a significant increase in MDA level (116.1%) with a decrease SOD activity (47.3%) at 16 weeks after ovariectomy (Muthusami et al., 2005; Cao et al., 2014; He et al., 2017; Zhou et al., 2021).

The biochemical and gene expression analyses in this study were confirmed *via* histological and histomorphometric analyses. Expected histological alterations were detected both in cancellous and cortical bone of OVX rats. The mean area of trabecular bone density and the thickness of the cortical bone was decreased (*p* < 0.05) in OVX compared to control rats. Additionally, the total number of osteocytes in the cancellous and cortical bones was significantly decreased in OVX compared to control rats. These findings were also reported by other studies (Park et al., 2008; Kalleny, 2011). Furthermore, osteoblast cells were absent on the endosteal surface, with a decrease (*p* < 0.05) in the number of osteocytes and a decrease in osteoclasts on the eroded surface of the bone. The decrease of estrogens after ovariectomy might explain the appearance of many resorption vacuoles in the bony lamellae or erosion on the endosteal surface of the bone as estrogen enhances the proliferation of osteoblasts and reduces the generation and life span of osteoclast (Inada and Miyaura, 2010).

The currently recognized relationship between osteoporosis and angiogenesis is that after ovariectomy, angiogenesis and the number of local blood vessels are reduced in the bone resulting in the OP (Wei et al., 2012), while an increase in local angiogenesis can alleviate osteoporosis (Tan et al., 2015). VEGF is a key mediator of angiogenesis as it attracts both endothelial and osteoprogenitor cells and stimulates them to produce trophic factors that enhance their survival and differentiation (Beamer et al., 2010; Zhang et al., 2020). In the current study, the abundance of VEGF and PCNA protein decreased significantly in femur bones from OVX rats compared to femur bones from control rats, indicating suppressing bone remodeling by inhibiting osteoblast differentiation and proliferation, which may be due to an increase in ROS levels. This is also confirmed in our histological findings as we recorded the absence of osteogenic and osteoblast lining on the endosteal surface.

Impairment in bone mineralization in the OVX rats was confirmed by the significant reduction of both femur cortical and trabecular BMDs by 36.8 and 26.1%, respectively. The rate of bone loss in the cortical bone is much lower and slower than in the trabecular bone (Srivastava et al., 2008). The higher sensitivity of the cortical bone in both OVX and treated rats compared to the trabecular bone in this study could be attributed to the faster growth rate of trabecular connectivity in the young rats aged less than 6 months (Liu et al., 2015). Additionally, it is concluded that the largest mean absolute bone loss occurs in the cortical compartment, but only during the first 60 days of age. With a longer study duration of 90 days, loss of trabecular bone may become more prominent (Srivastava et al., 2008) (Cervinka et al., 2013).

Compared to the OVX group, all treatment groups (OVX/E, OVX/ZnHA, and OVX/E + ZnHA) have lower values for ALP and PINP activity, with values for rats in OVX/E2+ZnHA group being closest to values for control rats. Coinciding with our results, ossein hydroxyapatite lowered PINP, and ALP also decreased by 46.7% (Albertazzi et al., 2004). In addition, treatment with nHA, nCh/HA, or nAg/HA reduced serum BALP levels significantly, according to a previous report (Fouad-Elhady et al., 2020b). E2 and/or ZnHA-NPs administration were also effective in restoring concentrations of Ca and P to normal levels compared to those for OVX rats. This increase in the OVX/E group may be connected to estrogen’s previously noted involvement in boosting Ca absorption (Hassan et al., 2016; Said et al., 2020). While the increase of Ca and P levels in the OVX/ZnHA group could be because HA is chemically related to the inorganic component of a bone matrix with the formula of Ca10(OH)2(PO4)6 (Kee et al., 2013). It has been noted that combined treatment in group OVX/E + ZnHA restored Ca and P to near the values for control rats, despite using half doses of each treatment in this group. In general, our result provides evidence that administration of ZnHA-NPs alone or with E2 was more effective than treatment with E2 alone on bone turnover markers (Ca, P, ALP, and PINP levels).

On the other hand, the OVX/E2 and OVX/E2+ZnHA treated groups had lower femoral estrogen receptors (ER-α and ER-β) mRNA with increased OC expression compared to OVX rats, indicating increased bone mineralization, even though rats in group OVX/ZnHA had greater OC mRNA expression than the OVX group, but the difference was not significant (*p* > 0.05). In addition, all treated groups exhibited higher levels of serum E2 compared to the OVX group. Coinciding with other findings, downregulation of ER-β in local bone tissue was positively correlated with estrogen dosage and improved angiogenesis (Zhang et al., 2019). The capacity of ZnHA-NPs to enhance serum estradiol levels in the current investigation suggests that zinc may have a role in the secretion of estrogen from glands other than the ovary (Bhardwaj et al., 2016). Administration of E2 and/or ZnHA-NPs resulted in decreased expression of estrogen receptors as compared to the OVX group.

Administration of E2 and/or ZnHA-NPs resulted in significant downregulation in RANKL and elevation in OPG expression in the femur, resulting in suppression of osteoclast genesis and decreased bone resorption in OVX/E2, OVX/ZnHA, and OVX/E2+ZnHA groups. This indicated that ZnHA-NPs might possess an anti-inflammatory effect owed to dipping zinc particles in HA. Our findings are parallel to previous reports that investigated estrogen at three different doses (5, 10, and 50 ug/kg/time) and used nHA, nCh/HA, or nAg/HA in treatments of osteoporotic rats (Zhang et al., 2019) (Fouad-Elhady et al., 2020b). Moreover, HA decreases the expression of NF-κB signaling in rat glioma C6 cells and human glioma U87MG ATCC cells (Guo et al., 2019).

Administration of E2, in the current study, has a powerful effect on the suppression of these cytokine levels as compared to those of the OVX group. This suggests the protective effect of estrogen on the bone because E2 is known to be involved in the suppression of inflammatory cytokines, which increase osteoclast genesis and bone resorption (Sunyer et al., 1999) and can selectively modulate some IL-1β receptor isoforms (IL-1R) in osteoclasts (Slemenda et al., 1997). The decline in levels of TNF-α, IL-1β, and IL-6 in rats treated with ZnHA may be attributed to zinc incorporated in nanoparticles (Ganesan et al., 2005). In addition, high zinc concentration reduced pro-inflammatory mediators (TNF-α, IL-6, and C-reactive protein) by inhibiting the IκB kinase (IKK)-α/NF-κB signaling pathway (Prasad et al., 2011). Combined treatment of E2 and ZnHA-NPs was more effective in the suppression of cytokine levels instead of using each alone in higher doses, and that is a good step toward using estrogen in low doses in the long run with ZnHA-NPs in the prevention or treatment of osteoporosis.

Estrogens have been shown to have antioxidant properties (Shihab et al., 2009). Our results revealed that E2 treatment elevated the GSH and SOD activities and decreased the levels of MDA and NO in serum compared to the OVX group; this is likely attributable to the phenolic structure of E2 that is comparable to that of well-known lipophilic antioxidants (Shihab et al., 2009). On the same line, E2 administration increased SOD and glutathione peroxidase expression (Borrás et al., 2003). Additionally, a substantial decrease in serum MDA and bone NADPH oxidase 4 expression, as well as an increase in SOD activity in both serum and bone tissue, was detected in OVX rats treated with 17-estradiol (25 μg/kg/day) for 16 weeks (Cao et al., 2014). However, in the present study, there were no significant changes in the levels of oxidative markers and antioxidants in OVX/ZnHA and OVX + E2+ZnHA treated compared to OVX rats, except that NO levels were significantly lower for rats in the OVX + E2+ZnHA group than the OVX group.

The histological alterations both in cancellous and cortical bone were restored by E2 and ZnHA-NPs administration. On the other hand, the abundance of VEGF and PCNA protein increased significantly in response to E2 and ZN HA supplementation. The increase in osteoblast proliferation and differentiation in the OVX/E2, OVX/ZnHA, and OVX + E2+ZnHA groups was the reason for reestablishing the expression of both VEGF and PCNA protein in the femur bone. The significant increase in collagen density in all treated groups in the current study indicates an enhancement of osteoblast activity and their maturation, synthesis of organic bone matrix components, and bone mineralization (Nam et al., 2016; Alippe et al., 2017; Oliveira et al., 2017).

Nanoparticles had been chosen to be involved in our study from the point of their rapidly growing concepts in tissue engineering for bone regeneration (Mirzaei and Darroudi, 2017) due to their ability to penetrate smaller blood capillaries and be absorbed by the cells, allowing drug delivery to the target sites (Li et al., 2020). Zinc is an essential trace element naturally located at sites of tissue calcification, and as bone mineralization increases, ALP uses zinc as a cofactor involved in bone mineralization (Anderson, 2003). Therefore, this could account for the superiority of ZnHA-NPs to preserve both femoral cortical and trabecular BMD over E2 replacement therapy. Additionally, in the combination group, a synergistic effect of ZnHA-NPs with E2 replacement therapy was sufficient in maintaining BMD comparable to that of control rats.

Although the present study discloses for the first time the effects of estrogen and ZnHA-NPs combined treatment of osteoporosis in the rat models, there are some limitations of the present work. Cell viability and hemolysis ratio following *in vitro* study with Zn-hydroxyapatite are needed as an indicator of treatment safety. The respective benefits of estrogen and/or ZnHA-NPs treatment in non-ovariectomized rats are also needed. A full exploration of the ZnHA-NPs extraosseous effects on tissues is required, particularly hepatic, cardiac, and renal tissues. The evaluation of parathyroid hormone and fibroblast growth factor 23 levels following the administration of estrogen, ZnHA-NPs, and their combination is considered another limitation in our study. In addition, to improve therapeutic impact, dose and treatment length must also be investigated further.

## Conclusion

The current study clearly indicates that ZnHA-NPs are an effective nano-biomaterial for the prevention and treatment of osteoporosis. The superior impact of ZnHA-NPs is due not only to its nano-size and form, which closely resembles that of HA crystals in natural bone, but also due to zinc particles, which enhance HA’s properties, especially its anti-inflammatory activity. Combined administration of ZnHA-NPs and E2 in low dosages has a promising potential efficacy in the prevention and treatment of osteoporosis, implying possible synergism between the two treatments. In addition, research on the effect of this combination on non-skeletal tissues is required. In order to improve therapeutic impact, dose and treatment length must also be investigated further.

## Data Availability

The original contributions presented in the study are included in the article/Supplementary Materials; further inquiries can be directed to the corresponding authors.

## Abbreviations

ALP, alkaline phosphatase; BMD, bone mineral density; CAT, catalase; Ca, calcium; ESR1, estrogen receptor alpha; E2, estradiol; ESR2, estrogen receptor beta; GSH, reduced glutathione; IL-1B, interleukin-1β; IL-6, interleukin-6; MDA, malondialdehyde; NO, nitric oxide; OP, osteoporosis; OPG, osteoprotegerin; OVX, ovariectomized; P, phosphorus; PCNA, proliferating cell nuclear antigen; PINP, pro-collagen type 1; RANKL, receptor activator of nuclear factor-kappa B (NF-kB) ligand; SOD, superoxide dismutase; TNF-α, tumor necrosis factor-alpha; VEGF, vascular endothelial growth factor; ZnHA-NPs, nano zinc hydroxyapatite.
